# A Web-Based Public Health Intervention to Reduce Functional Impairment and Depressive Symptoms in Adults With Type 2 Diabetes (The SpringboarD Trial): Randomized Controlled Trial Protocol

**DOI:** 10.2196/resprot.7348

**Published:** 2017-08-03

**Authors:** Judy Proudfoot, Janine Clarke, Jane Gunn, Susan Fletcher, Samineh Sanatkar, Kay Wilhelm, Lesley Campbell, Nicholas Zwar, Mark Harris, Helen Lapsley, Dusan Hadzi-Pavlovic, Helen Christensen

**Affiliations:** ^1^ Black Dog Institute Randwick Australia; ^2^ School of Psychiatry University of New South Wales (UNSW) Sydney Sydney Australia; ^3^ Department of General Practice University of Melbourne Melbourne Australia; ^4^ Diabetes and Metabolism Division Garvan Institute of Medical Research Sydney Australia; ^5^ School of Medicine University of Wollongong Wollongong Australia; ^6^ Centre for Primary Health Care and Equity University of New South Wales (UNSW) Sydney Sydney Australia

**Keywords:** type 2 diabetes, depression, Web-based intervention

## Abstract

**Background:**

Depressive symptoms are common in people with type 2 diabetes and contribute to adverse health consequences that substantially impact social and vocational function. Despite the existence of effective depression treatments, the majority of people with type 2 diabetes do not access these when needed. Web-based alternatives to more traditional psychotherapies offer a potential solution to reducing the personal and economic burdens of type 2 diabetes.

**Objective:**

This paper outlines the protocol for a randomized controlled trial (RCT) of myCompass, a Web-based public health psychotherapy intervention, in people with type 2 diabetes. Fully automated, interactive, and delivered via the Internet without clinician support, myCompass teaches cognitive behavioral therapy-based skills and supports symptom monitoring to improve daily functioning and reduce mild-to-moderate mental health symptoms.

**Methods:**

A two-arm RCT will be conducted. People with type 2 diabetes and mild-to-moderately severe depressive symptoms will be recruited from the community and general practice settings. Screening and enrollment is via an open-access website. Participants will be randomized to use either myCompass or an active placebo program for 8 weeks, followed by a 4-week tailing-off period. The placebo program is matched to myCompass on mode of delivery, interactivity, and duration. Outcomes will be assessed at baseline and at 3-month, 6-month, and 12-month follow-up. The primary study outcome is work and social functioning. Secondary study outcomes include depressive and anxious symptoms, diabetes-related distress, self-care behaviors, and glycemic control.

**Results:**

Nationwide recruitment is currently underway with the aim of recruiting 600 people with type 2 diabetes. Recruitment will continue until October 2017.

**Conclusions:**

This is the first known trial of a Web-based psychotherapy program that is not diabetes specific for improving social and vocational function in people with type 2 diabetes and mild-to-moderately severe depressive symptoms. With the increasing prevalence of type 2 diabetes and depression, a potentially scalable public health intervention could play a very large role in reducing unmet mental health need and ameliorating the personal and societal impact of illness comorbidity.

**Trial Registration:**

Australian New Zealand Clinical Trials Registry (ANZCTR) Number: ACTRN12615000931572; https://www.anzctr.org.au/Trial/Registration/TrialReview.aspx?id=368109 (Archived by WebCite at http://www.webcitation.org/ 6rh3imVMh)

## Introduction

### Background

Type 2 diabetes is a common chronic and disabling disease and a major contributor to global disease burden [1]. Depression is frequently comorbid with type 2 diabetes and contributes independently to a range of adverse health outcomes that substantially compromise social and vocational functioning. These include greater diabetes symptom burden, poorer self-care, and increased risk of micro- and macrovascular complications and mortality [2]. In addition, the economic impact of disease comorbidity is considerable with functional disability, functional dependence, workplace productivity losses, health service use, and health care costs higher for people with both conditions than those with diabetes alone [2,3].

Research supports a bidirectional relationship between type 2 diabetes and depression [4]. Findings supporting increased risk of type 2 diabetes in people with depression [5] are generally explained with reference to physiological (eg, hypothalamic- pituitary-adrenal axis dysfunction [6]), motivational (eg, poorer self-care, adiposity, and inactivity [6,7]), and/or pharmacologic (eg, impact of antidepressant medication on glycemic control [5]) factors. At the same time, increased risk of depression in people with type 2 diabetes is generally attributed to the emotional burden of living with a complex and demanding disease that often intrudes into normal lifestyle [8]. The likelihood of a reciprocal relationship between type 2 diabetes and depression makes the personal and societal impact of illness comorbidity potentially immeasurable [4].

Despite the existence of evidence-based therapies for depression in diabetes, including cognitive behavioral therapy (CBT) and antidepressant medication, upward of 60% of people with comorbid conditions do not receive mental health treatment [9]. In the primary health care setting, where most people with type 2 diabetes access medical treatment, low screening rates mean that depressive symptoms are often missed [9] and only a minority of patients who screen positive accept a referral for face-to-face support [10]. At the same time, personal, social, and structural barriers to seeking help—including privacy concerns and stigma, lack of support and poor relationships with health care providers, time and lifestyle constraints, financial cost, and lack of service availability—compromise access to satisfactory mental health care for many patients [11,12]. There is considerable opportunity, therefore, to reduce the personal and societal burden of illness by facilitating greater access to more flexible and cost-efficient mental health treatments for this patient group.

Internet delivery of evidence-based psychological therapies is a popular, clinically effective, and cost-effective means of increasing treatment reach; a small number of Web-based diabetes-specific interventions that directly target depression have been evaluated [13]. These include van Bastelaar et al’s [14] adaptation of Lewinsohn’s Coping with Depression Program and Nobis et al’s [15] application of systematic behavioral activation and problem-solving therapy (ie, GesundheitsTraining.Online Mood Enhancer Diabetes [GET.ON MED]). Findings from these studies establish the symptom benefits of Internet-delivered self-help for diabetes patients with clinically relevant levels of depressive symptomatology. However, subthreshold depression is more prevalent in type 2 diabetes than clinical depression and is associated with increased functional limitation and disability, including reduced social and vocational performance [16,17]. As such, the effectiveness of electronically delivered psychotherapy as a treatment approach for type 2 patients with mild-to-moderate depressive symptoms also warrants rigorous scientific attention.

Proudfoot et al [18] have previously published controlled data showing that mild-to-moderate mental health symptoms— including symptoms of depression, anxiety, and stress—are reduced following use of the broadly available Web-based program, myCompass, with treatment benefits extending to work and social functioning. More recently, in an uncontrolled pilot study, myCompass showed promise as an acceptable and effective treatment for depression and functional disability in people with type 1 and type 2 diabetes [19]. myCompass differs from the interventions described in van Bastelaar et al [14] and Nobis et al [15] in that it is a fully automated (ie, no therapist input) public health program that is generic in its therapeutic content (ie, not diabetes sensitive). An intervention approach that is capable of treating depressive symptoms without disease-specific modification is potentially a more efficient and accessible alternative to meeting the unmet mental health needs of people with type 2 diabetes. Generic skills may also assist the increasing number of individuals experiencing multi-morbidity, for whom depression co-occurs with somatic symptoms of multiple illnesses (eg, diabetes, heart disease, hypertension, and kidney disease) [20,21]. From a primary care perspective, a public health program may have important pragmatic advantages in the primary care setting, where time pressures often impede dissemination of, and prohibit practitioner training in, multiple disease-specific tools, and where treatment of multi-morbidity and undifferentiated physical and mental health symptoms is particularly relevant.

### Objectives

This paper was prepared using the Standard Protocol Items: Recommendations for Interventional Trials (SPIRIT) guidance for presenting clinical trial protocols [22] and the Consolidated Standards of Reporting Trials of Electronic and Mobile HEalth Applications and onLine TeleHealth (CONSORT-EHEALTH) checklist [23]. The paper describes a randomized controlled trial (RCT) of the Web-based intervention, myCompass, in patients with comorbid type 2 diabetes and mild-to-moderately severe depressive symptoms. The primary aim of this RCT, called SpringboarD, is to determine whether treatment with myCompass improves work and social function for people with type 2 diabetes and mild-to-moderate depressive symptoms. As functional disability predicts further functional deterioration, functional dependence, and increased use of health services and health care costs [16], depression treatments that improve work and social functioning may substantially reduce the personal and economic burden of comorbid depression and type 2 diabetes. Specifically, we hypothesize that the intervention group will show significant improvement in self-reported functioning socially and in the workplace compared with a placebo-controlled condition.

Our secondary aim is to evaluate the impact of myCompass on a range of symptom- and disease-related variables known to impact a patient’s disease management and blood glucose control. Specifically, in addition to depressive symptoms, we will examine whether myCompass is more effective than a placebo condition in improving anxiety symptoms and diabetes-related distress. Defined as a person’s emotional adjustment to the chronic stressors specific to diabetes, diabetes-related distress is of particular interest due to its greater prevalence and potential role in mediating the relationship between depression and glycemic control [24]. While we have preliminary evidence suggesting that diabetes-related distress may improve following treatment with myCompass [19], replication of this finding in a controlled study is required.

### Trial Design

The study is designed as a two-arm individually randomized RCT and is conducted entirely online. Participants allocated to the intervention group have full access to the myCompass program for 8 weeks, followed by a 4-week tailing-off period to facilitate maintenance. Participants randomized to the control group have access to a placebo Internet-delivered program that is matched to myCompass on mode and duration of delivery and interactivity, but is void of therapeutic content. Participants in both groups have uninterrupted access to treatment as usual for their diabetes during the study.

## Methods

### Participants, Interventions, and Outcomes

#### Study Setting

The setting for this study is Australia. An estimated 1 million Australian adults—5% of the population—had self-reported type 2 diabetes in September 2016, and rates are similar across metropolitan, regional, and remote areas [25].

#### Eligibility Criteria

This study focuses on adults with type 2 diabetes and mild-to-moderately severe depressive symptoms. People are eligible to take part if they are aged 18-75 years, screen positive for depression on the 2-item Patient Health Questionnaire (PHQ-2) (ie, score ≥2) [26], and have access to an Internet-enabled device (eg, computer, tablet, and/or mobile phone). People who screen positive for depression complete the full 9-item Patient Health Questionnaire (PHQ-9) at screening so that the level of symptom severity can be determined.

Exclusion criteria include inability to read English with ease, severe depressive symptoms on the full PHQ-9 (ie, score >19), probable psychosis as measured by the psychosis screener developed for the Australian National Mental Health and Wellbeing Survey [27], currently receiving face-to-face counseling or therapy for depression, changed antidepressant medication in the previous 2 months, high suicide risk as assessed by item 9 of the PHQ-9, and previous use of the myCompass program.

#### Interventions

##### Active Intervention

**Figure 1:**
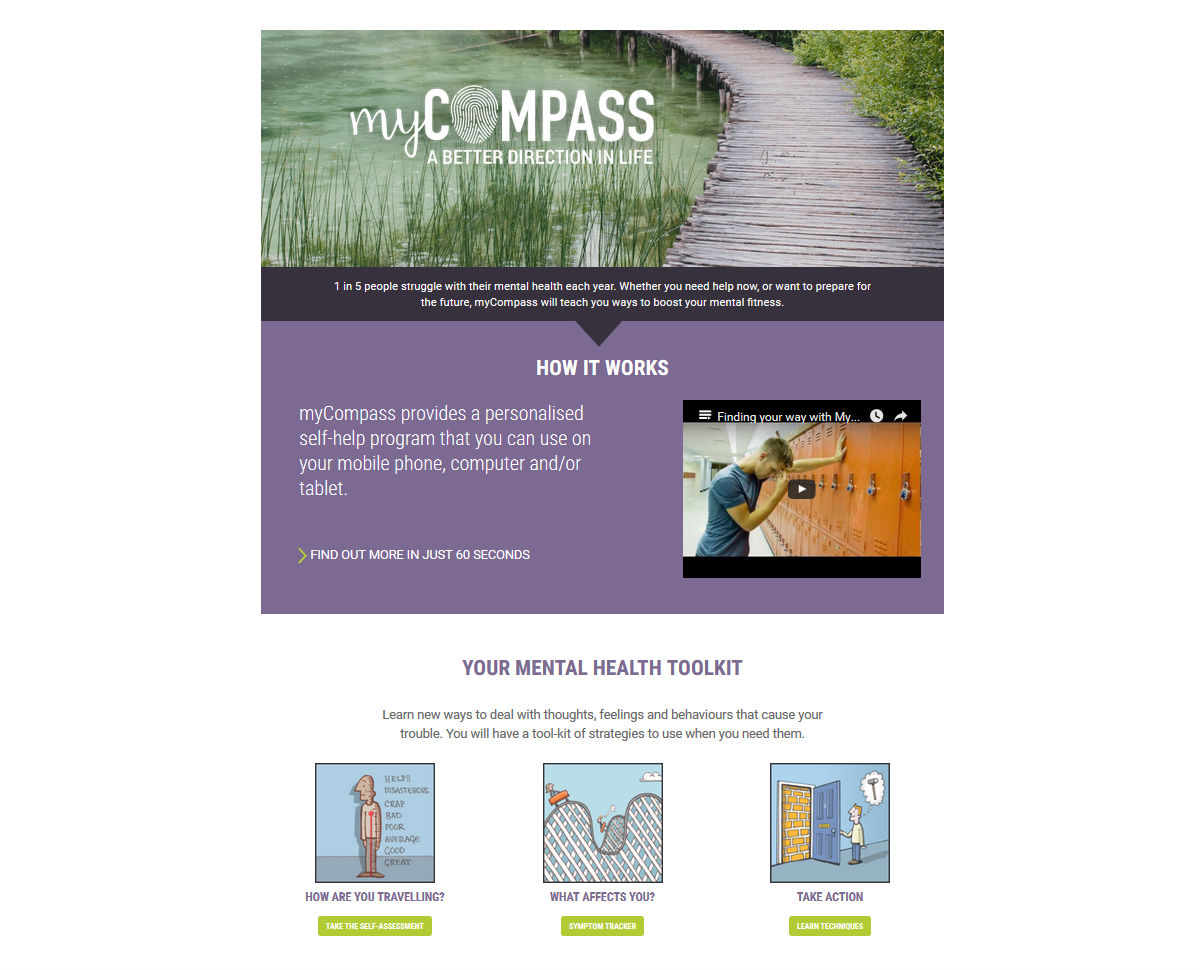
Screenshot of myCompass landing page.

**Figure 2:**
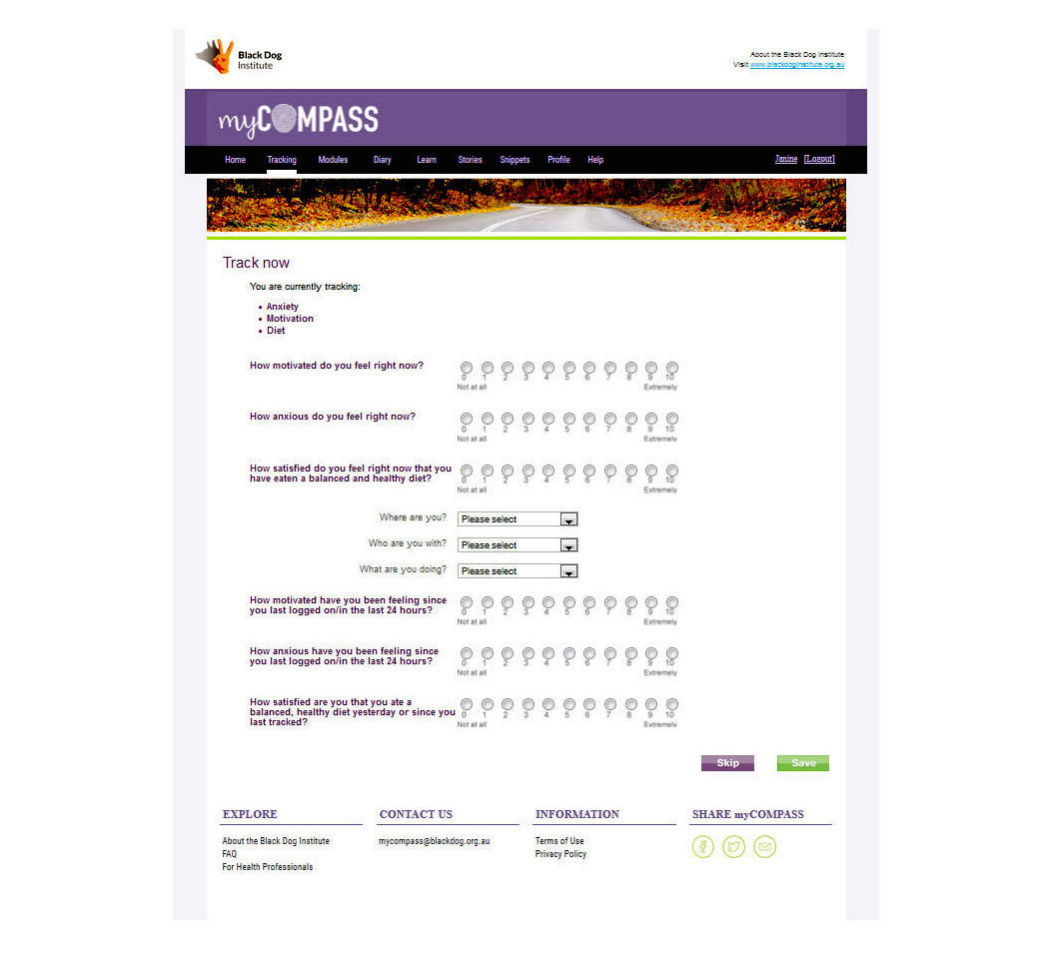
Screenshot of myCompass self-monitoring page.

**Figure 3:**
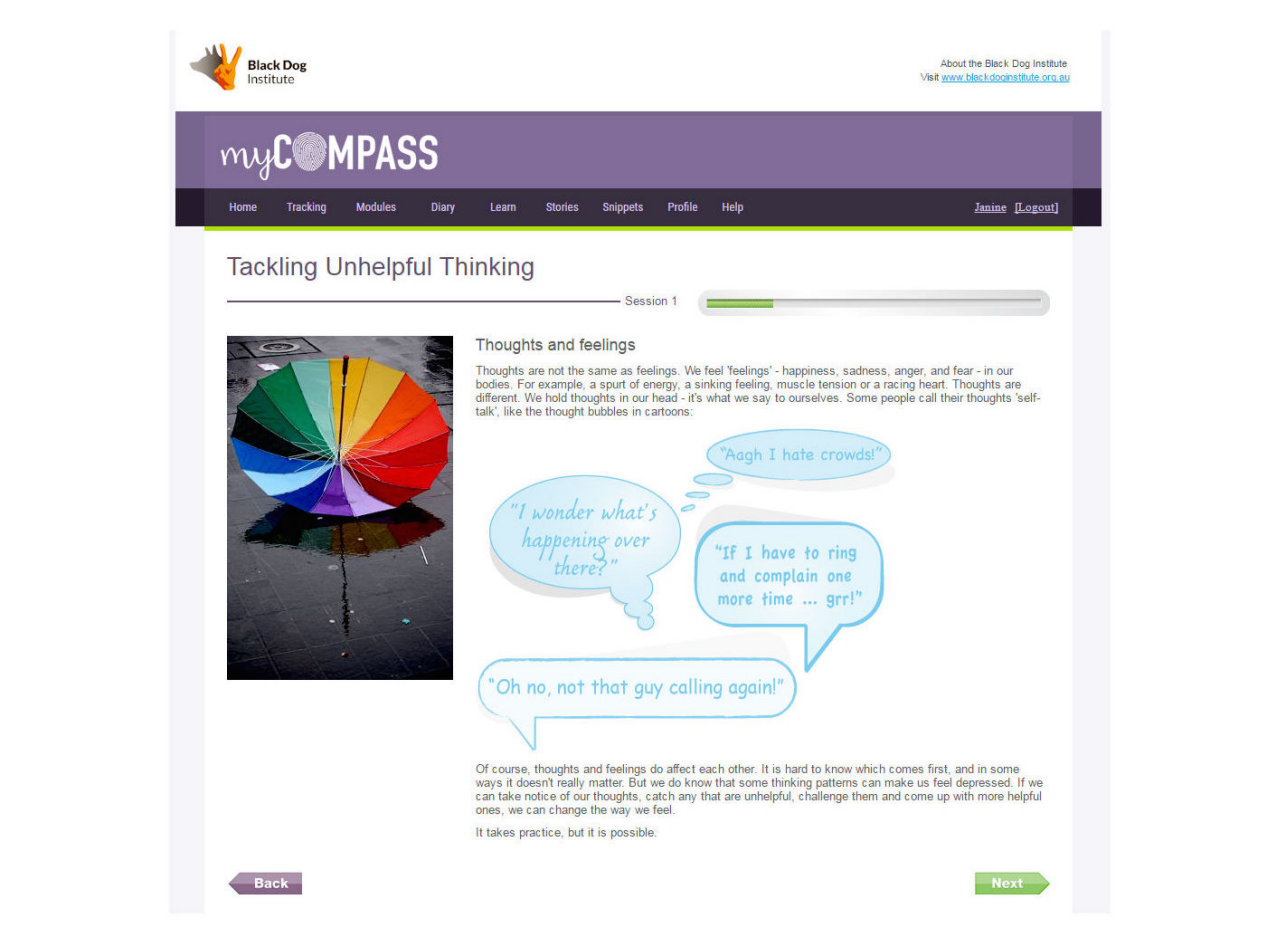
Screenshot of a page from the myCompass module Tackling Unhelpful Thinking.

**Figure 4:**
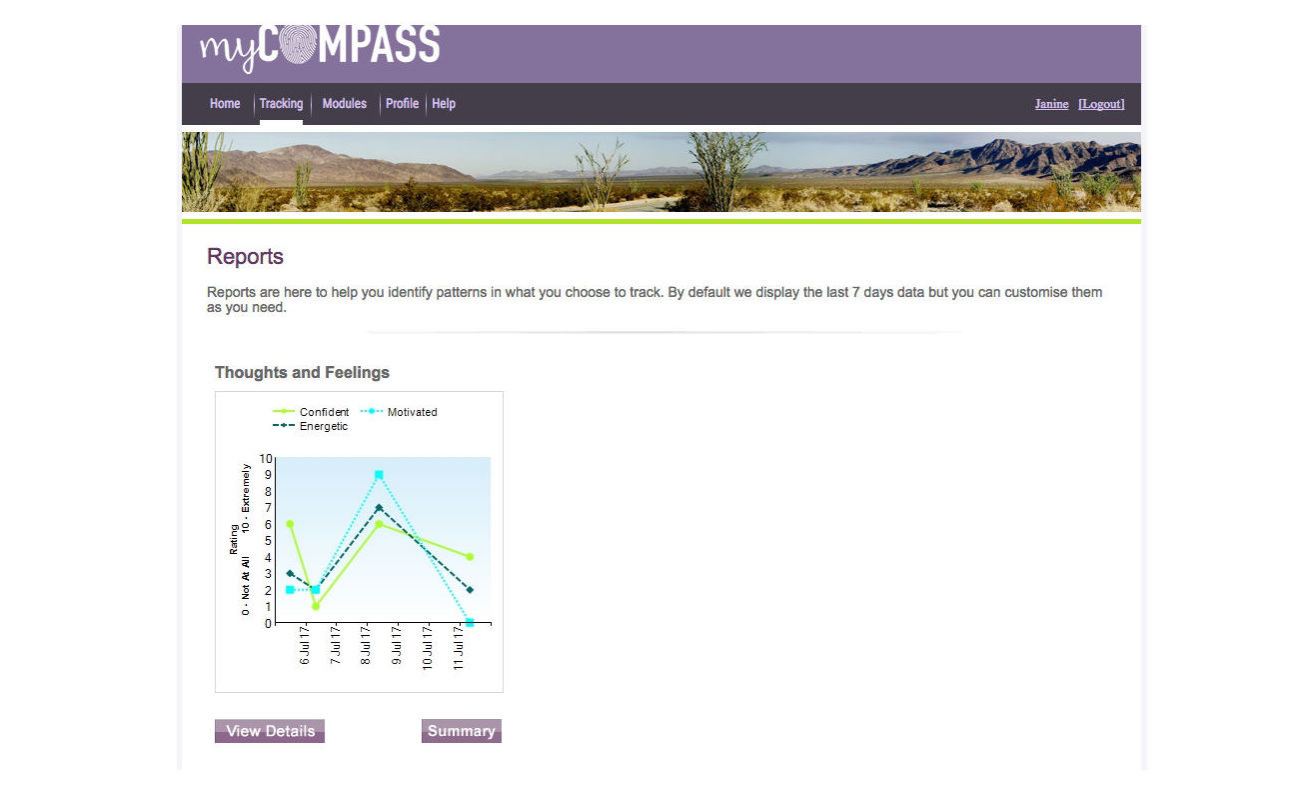
Screenshot of myCompass graphical feedback.

The active intervention, myCompass [28], is a fully automated, self-help, public health intervention that is tailored to the user and has no therapist involvement in its delivery. Program tailoring occurs via users’ responses to a symptom profiler completed at registration. In-built algorithms target the user’s most salient symptoms and provide recommendations about the symptoms and/or behaviors they might consider monitoring and the treatment modules likely to be of greatest therapeutic benefit. There is flexibility, however, for users to choose their own set of self-monitoring dimensions and treatment modules (see Figures 1-4).

Self-monitoring of symptoms and lifestyle factors occurs in real time via mobile (eg, phone or tablet) and stationary (eg, desktop computer) devices. Users can self-monitor up to three symptoms and behaviors of their choice at any one time—selected from a list of 20—or three that are recommended to them by the program. Each symptom is rated on a 10-point scale. At the time of rating, users also provide contextual information about where they are, what they are doing, and who they are with using a series of drop-down menus. Users can schedule short message service (SMS) text message or email reminders to facilitate self-monitoring—frequency of reminders is determined by the user; receive and print graphical feedback about their monitoring, including contextual information, on their phone or computer to monitor change and assist identification of triggers; and elect to receive helpful facts, mental health care tips, or motivational statements by SMS text message or email.

myCompass treatment modules were developed by mental health professionals at the Black Dog Institute in Sydney, Australia. The program contains 14 skill-building modules derived from CBT, interpersonal psychotherapy, problem-solving therapy, and positive psychology that are interactive and available for completion on computer and tablet devices. Module content covers topics such as *Managing Fear and Anxiety*, *Tackling Unhelpful Thinking*, *Managing Loss and Major Life Change*, and *Solving Problems*. Each module comprises three 10-minute sessions and includes home practice tasks for completion between the weekly sessions to promote skill generalization. The module targeting stress in diabetes has been deactivated for trial participants, to ensure that the intervention is a generic public health program. Participants are encouraged to complete at least three modules during the intervention period, during their own time.

User privacy is managed by a password-protected log-on and by ensuring that user-generated data (ie, self-monitoring ratings) are not stored on the user’s phone, but are instead transferred via the Internet using Secure Sockets Layer protocol, which encrypts transmitted data by rendering it unreadable to anyone other than the intended recipient, and by storing the data in secure servers. The data is reidentifiable only with the list of study participant codes, to which only the named researchers will have access and which will be stored in a password-protected file separate from the study data. Registering to use myCompass is free and users are not billed for the SMS text messages they receive.

Participants randomized to myCompass have access to the full program for 8 weeks, followed by a 4-week tailing-off period during which only the symptom-monitoring function will be accessible. Research has shown that adherence with online interventions is improved when users receive program feedback that is personalized in its content [29]. For this reason, myCompass users receive automated personalized feedback via email about their use of the program’s self-monitoring and module functions at fixed intervals (ie, weeks 1, 3, 5, and 7).

##### Placebo Intervention

**Figure 5:**
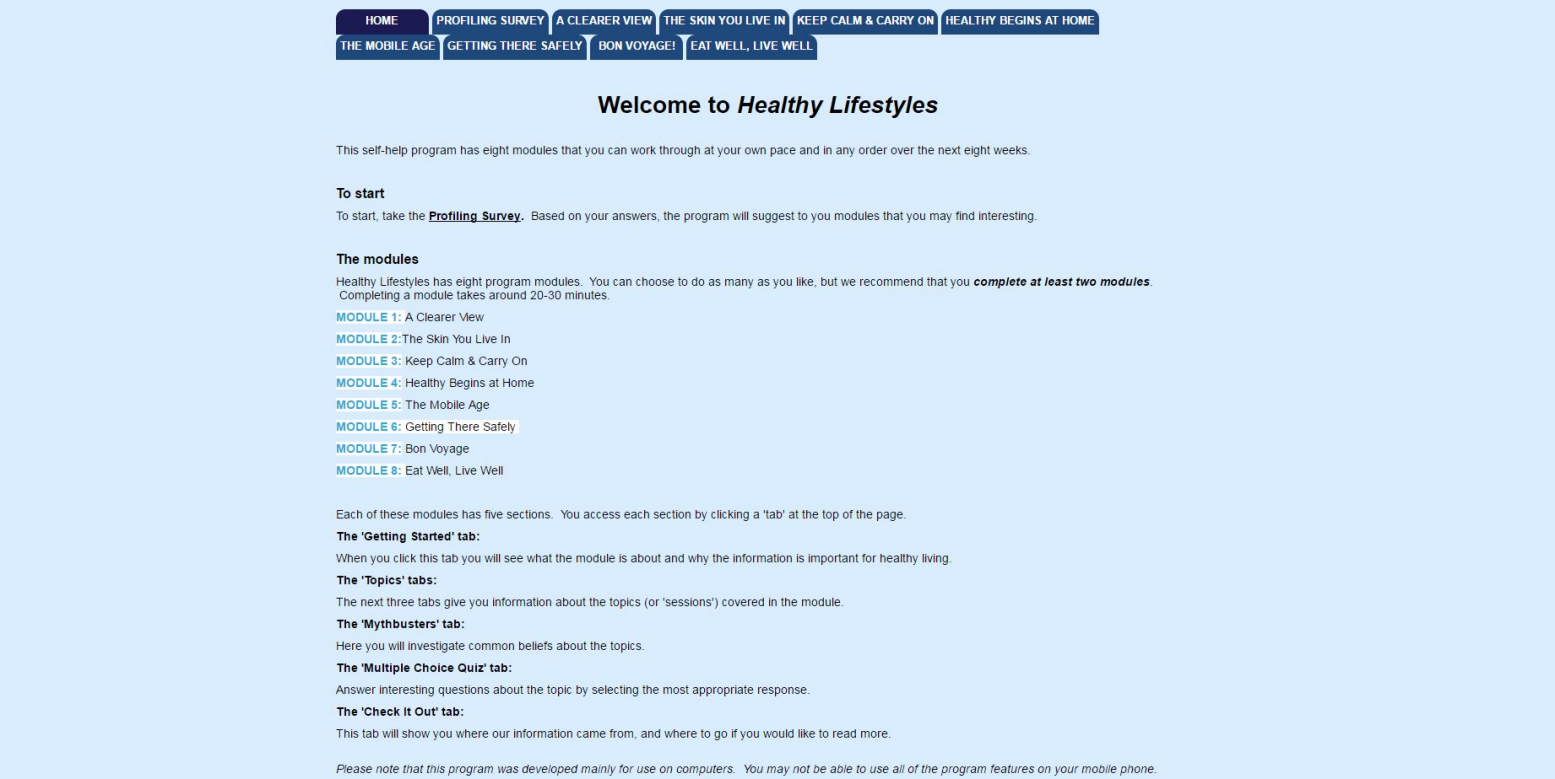
Screenshot of landing page for placebo program, Healthy Lifestyles.

**Figure 6:**
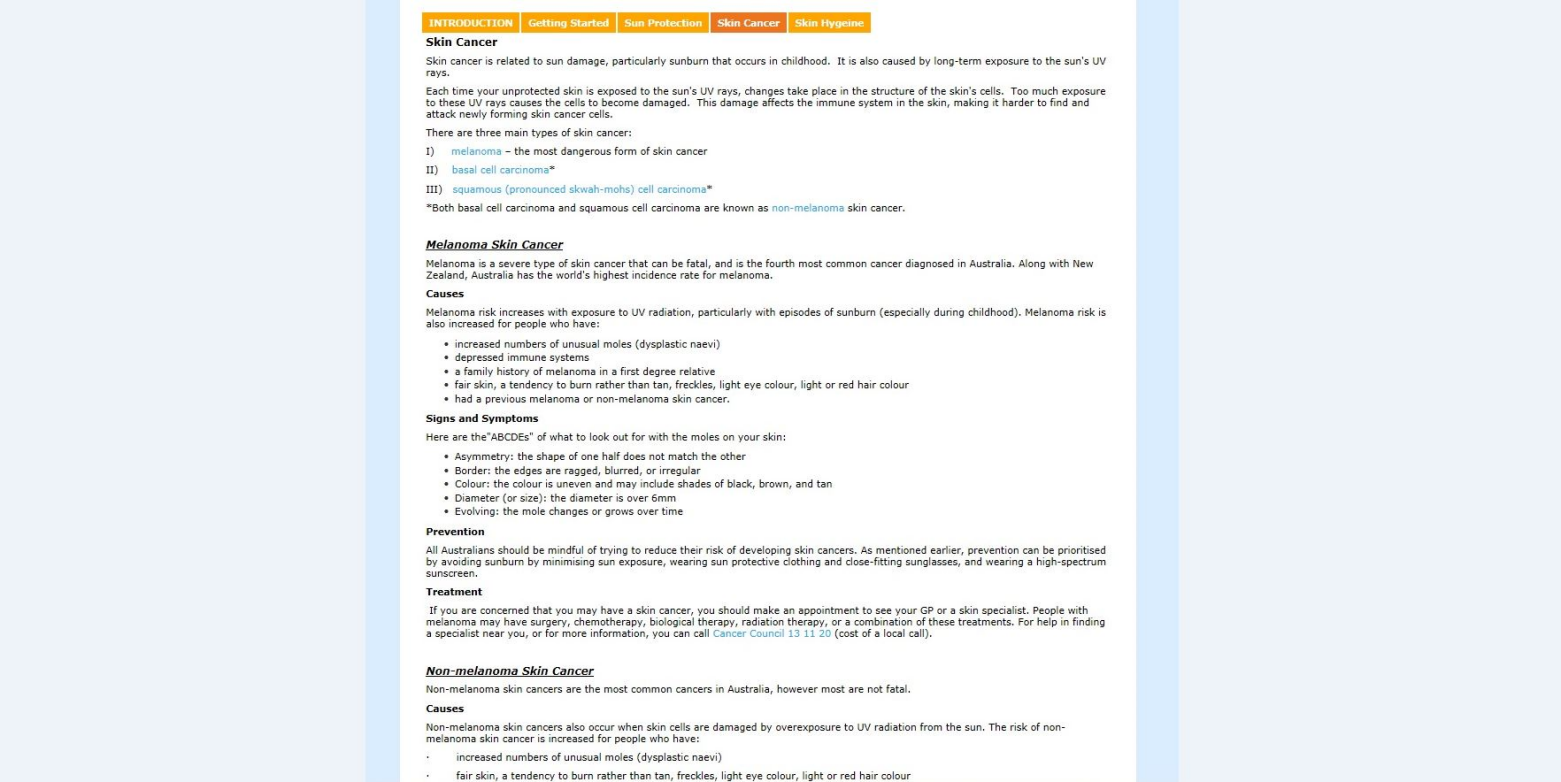
Page from the Healthy Lifestyles module, The Skin You Live In.

**Figure 7:**
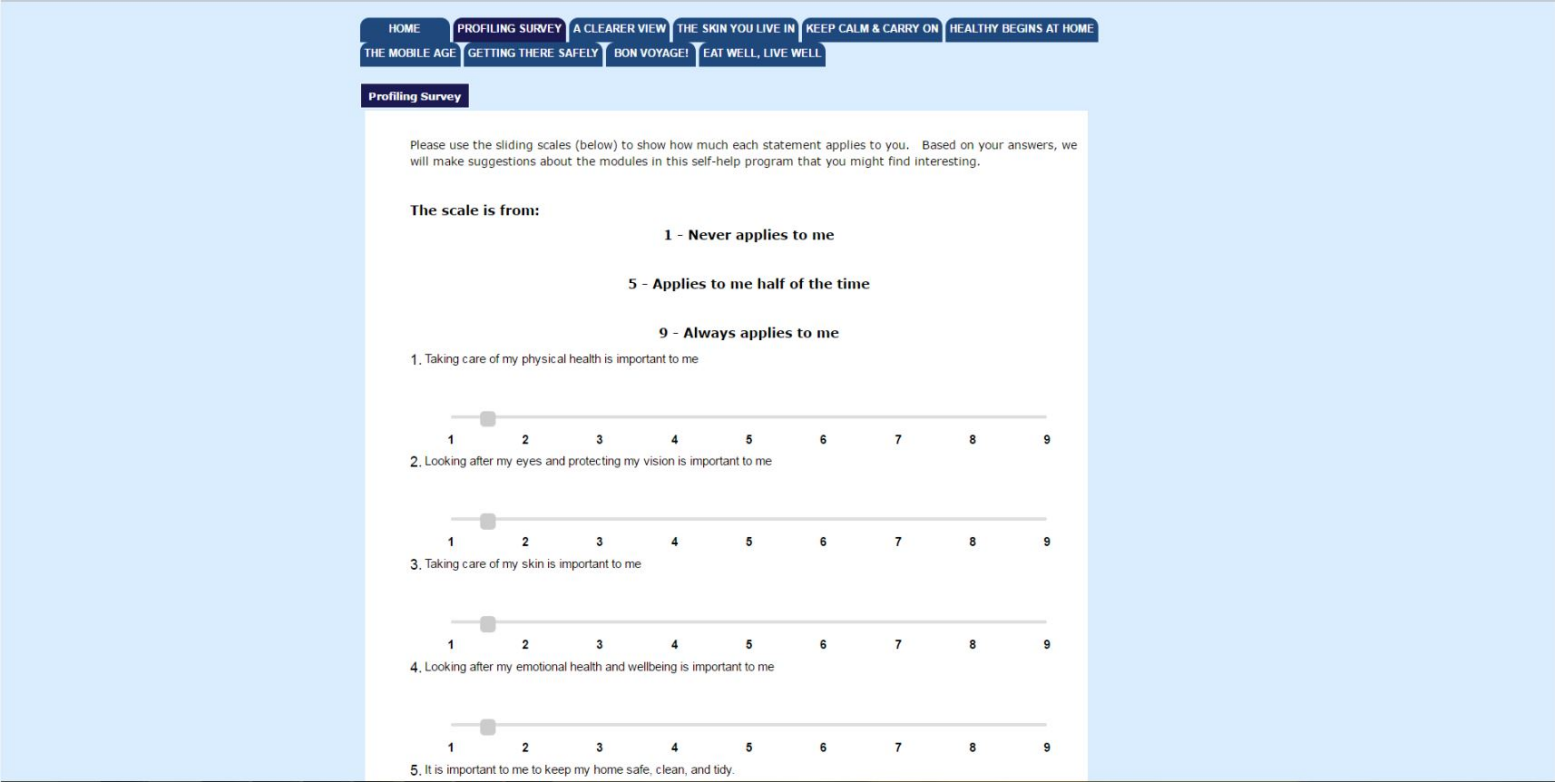
Sample interactive task from the Healthy Lifestyles module.

The placebo program, Healthy Lifestyles, was adapted from a control program used in previous studies by members of the research team. Like myCompass, the program offers users program tailoring at the outset, followed by access to a range of interactive modules containing health- and lifestyle-related information, including skin care and eye health. It contains no therapeutic content, has high face validity as a health and lifestyle intervention, and has previously shown no symptom impact [30]. Participants have full access to the placebo condition for 8 weeks, plus a 4-week tailing-off period (see Figures 5-7).

To further replicate the interactivity of myCompass, users of the placebo program receive an email at weeks 1, 3, 5, and 7 containing a brief reminder to log into the program, but no feedback about their use of the program. They also receive a weekly email containing a factual statement about a health or lifestyle issue.

**Table 1:** Assessments completed at assessment phases.

Assessments		Assessment phase			
		T1^a^	T2^b^	T3^c^	T4^d^
**Baseline**						
	Demographic data	X			
	Disease/treatment data	X	X	X	X
	Mental health history	X			
Primary outcome	Work and Social Adjustment Scale [31]	X	X	X	X
**Secondary outcomes**						
	Patient Health Questionnaire-9 [26]	X	X	X	X
	Diabetes Distress Scale [32]	X	X	X	X
	Generalized Anxiety Disorder Scale [33]	X	X	X	X
	Self-Management Profile for Type 2 Diabetes Scale (behavior items only) [34]	X	X	X	X
	Glycosylated hemoglobin (HbA1c)	X		X	X
	Days out of role [35]	X	X	X	X
	Health service utilization	X	X	X	X

^a^T1: baseline assessment and allocation to intervention or placebo group.

^b^T2: completion of intervention period and online postintervention assessment.

^c^T3: completion of 6-month follow-up assessment.

^d^T4: completion of 12-month follow-up assessment.

#### Outcomes

A summary of assessments completed online by participants at baseline, postintervention, and follow-up is presented in Table 1 [26,31-35].

The primary outcome is work and social functioning, which is measured by the Work and Social Adjustment Scale (WSAS) [31]. The WSAS is a psychometrically sound measure of the impact of mental health problems on daily functioning in five domains: work, social leisure activities, private leisure activities, home management, and personal relationships [31,36]. Scores range from 0 to 40, with higher scores indicating poorer adjustment.

Secondary outcomes include the PHQ-9, the Diabetes Distress Scale [32], the 7-item Generalized Anxiety Scale [33], and the Self-Management Profile for Type 2 Diabetes Scale [34].

At baseline and again at 6- and 12-month follow-up, glycosylated hemoglobin (HbA1c) data will also be collected from participants’ medical records, with their consent. The HbA1c test is a blood test showing a person's average blood glucose level over the previous 3 months and is measured as part of routine clinical care to monitor long-term blood sugar control in people with diabetes. In all cases, the most recent result will be obtained.

Additional measurements include collection of disease-related data (eg, age of onset and treatment regimen), sociodemographic data (eg, age, gender, education, and occupation), and mental health history data (eg, service use and previous diagnoses) at baseline. Service utilization and supports, including medication, received for problems related to mental health and diabetes are also assessed at each assessment point. Days out of role are measured using an item from the World Health Organization Disability Assessment Schedule that asks people to note the number of days in the previous 30 that they were completely unable to perform their work or normal activities because of problems with their physical or mental health [35].

At the conclusion of the trial, data indicating the extent of participant engagement with myCompass will be extracted from the program, including frequency of log-ins, number of modules started and completed, and self-monitoring frequency.

#### Participant Timeline

Participant consent, screening, and assessment takes place online via a secure study-specific website [37]. After providing informed consent, potential participants complete the online screening tool to determine eligibility. Unsuitable candidates receive automated feedback explaining the reason for their ineligibility; details of crisis supports and other self-help and face-to-face resources are provided to individuals, as appropriate.

**Figure 8:**
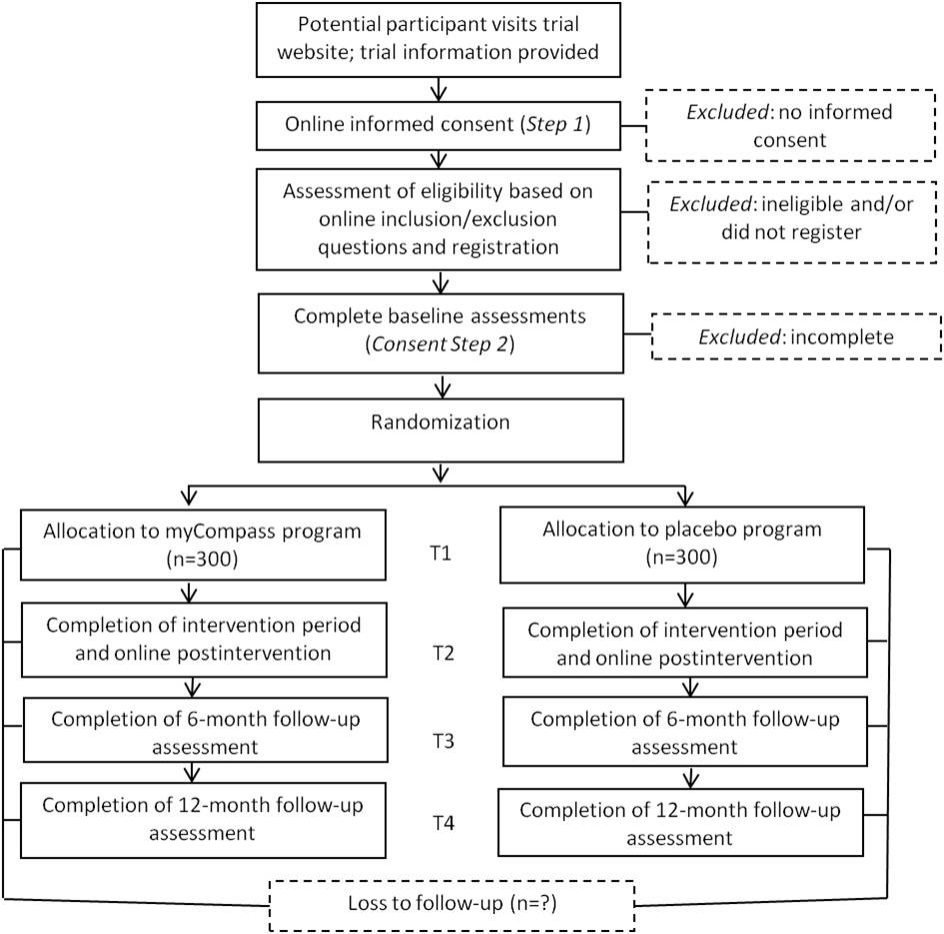
SpringboarD participant flow diagram.

Eligible candidates are registered in a secure Web-based platform where they complete the baseline questionnaire and are immediately allocated randomly to either the intervention program or control program for a period of 12 weeks. Subsequent assessment points coincide for both groups at postintervention (12 weeks) as well as 6 and 12 months after randomization (see Figure 8).

#### Sample Size

The primary study outcome is work and social functioning, which is measured by the WSAS [31]. In a previous study comparing myCompass with an active control intervention, Proudfoot et al [18] obtained an average between-group effect size—based on estimated marginal means—of Cohen *d*=0.3 for work and social functioning. We calculated the sample size for this study using a series of *t* tests (cross-sectional comparison of arms) with alpha=.05 and desired power=80%. The sample size required was N=350 (175 per arm).

We cross-checked the sample size calculation using a reduction of 5 points on our secondary outcome, the PHQ-9, and got a similar outcome. A 5-point reduction on the PHQ-9 is considered an adequate treatment response [26].

Previous studies indicate attrition rates of approximately 40% in eHealth studies, generally [38]. As such, we aim to recruit 300 participants in each arm of the study (ie, 600 in total) for sufficient statistical power for completer analyses.

#### Recruitment

Study participants are being recruited via promotional materials distributed in general practice settings in New South Wales and Victoria—where the majority of Australians with type 2 diabetes reside—and disseminated nationally via print advertisements; social media posts, including Facebook and Twitter; clinical research registries; and other publicity channels of state and local diabetes stakeholder groups and the Black Dog Institute.

Promotional materials invite interested candidates to visit the SpringboarD Project website [37] to provide consent and complete the screening tool. The recruitment message for the study focuses on learning new ways to deal with stress and distress and live active and emotionally healthier lives with type 2 diabetes. Recruitment has commenced and will continue until October 2017 or until our sample target is reached.

### Assignment of Interventions

#### Allocation

We are using computerized blocked randomization with blocks of eight to assign participants to the two treatment conditions. Randomization to the intervention and placebo program occurs immediately after a participant completes the baseline measures using an automated system built into the study software. In this way, the allocation sequence is applied without the researchers’ knowledge.

#### Blinding

The placebo program has been developed to replicate the mode of delivery, interactivity, and duration of myCompass and participants in each group will be treated equally by the research team. It is not possible, however, to assume that participants will remain blind to study allocation during the intervention and follow-up periods.

### Data Collection, Management, and Analysis

#### Data Collection

The majority of primary and secondary outcome data is being collected electronically via standardized self-report questionnaires that are completed by logging into the SpringboarD study website (described above). At each assessment point, the website sends a unique link to the study questionnaire via email. Questionnaire data is maintained on a secure server at the University of New South Wales (UNSW) Sydney and is downloaded periodically for storage in a password-protected data file accessible by two project personnel (JC and SS). HbA1c data is collected from each participant’s general practitioner (GP) via phone, mail, and/or facsimile.

#### Retention

Participant attrition is a major concern in studies of unguided interventions [38]. To facilitate trial retention, we are utilizing a combination of strategies covering various themes that have been used with success in previous studies. First, a systematic schedule of *push* messages, including personalized email and telephone prompts and reminders, is being implemented to motivate questionnaire completion at each of the postintervention and follow-up assessments [39]. Second, Aus $30 gift vouchers are provided to participants who return completed questionnaires at each time point to compensate them for the time and effort they have expended [40]. Third, a sense of project identity is maintained by using a study logo and design template (ie, set fonts, formatting, and colors) to create the SpringboarD *brand*, and by distributing quarterly newsletters to participants to update them on trial progress [41]. Finally, a phone call from the research team within a week of registration provides participants a minimum level of human contact and the team an opportunity to express thanks, provide encouragement, and confirm personal details [42].

#### Statistical Analysis

Participants in the intervention and control groups will be compared at baseline using chi-square tests for categorical data and *t* tests for continuous data to assess randomization success. Treatment effects on primary and secondary outcomes will be evaluated by intention-to-treat analysis using mixed-models repeated measures (MMRM). In MMRM, no participant is removed from the analysis because all available data are used to obtain parameter estimates. Effect size will be measured using Cohen *d*. For all outcome measures, within- and between-group differences will be standardized to Cohen *d* using the pooled standard deviation of the observed scores obtained at baseline. We plan to analyze contrasts between intervention and control groups at postintervention and at 3-month, 6-month, and 12-month follow-up.

Supplementary analyses will use data for completers and will also investigate whether there are any differences by recruitment source, duration of diabetes, and presence of comorbid conditions.

### Monitoring

The integrity of the trial, including data collection and monitoring, trial progress, adverse events, and compliance with UNSW Sydney Human Research Ethics Committee (HREC) reporting procedures, is overseen by the Steering Committee consisting of the chief (JP) and associate investigators. The Steering Committee will meet biannually over the lifespan of the project. Adverse events may include unfavorable changes to mental health or diabetes control and may be related or unrelated to the study. As the study does not impact routine diabetes care and is examining the effect of an evidence-based intervention for people with mild-to-moderately severe depression (ie, serious mental illness is an exclusion criteria), no serious adverse events are anticipated and no interim analyses are planned.

### Ethics and Dissemination

#### Research Ethics Approval

The SpringboarD study protocol and materials have been approved by the HREC at UNSW Sydney and registered with the Australia and New Zealand Clinical Trials Register (ACTRN12615000931572). Annual reports and substantive amendments to this protocol will be submitted to the HREC for approval by the chief investigator. The study coordinator (JC) is responsible for communicating protocol changes to relevant stakeholders, including the Australian New Zealand Clinical Trials Registry.

#### Consent or Assent

Information about the study is provided on the SpringboarD project website; individuals can choose to read the information online or download a PDF to keep. Consent is obtained online in a two-stage process. First, individuals consent to the study by checking a box at the end of the study information page and progressing to the page containing the eligibility screen. Eligible individuals are then provided the option of registering an account with the study website (ie, username and password); those who opt not to register are also considered to have not consented to the trial.

Participants consent to the project team informing their treating GP of his or her involvement in the study to facilitate HbA1c data collection; they also provide a point of emergency contact should a participant score in the severely distressed range of the PHQ-9 (ie, score >19) or be at risk of self-harm (ie, score 3 on item 9 of the PHQ-9) at any assessment point. GPs are informed by mail within 2 weeks of their patient’s enrolment, at which time they are requested to inform the research team if a diagnosis of type 2 diabetes has not been given.

#### Confidentiality

The eligibility screen is conducted anonymously such that no personal information about potential participants is collected. Only eligible individuals provide identifying information that is downloaded and stored separately from study data in a password-protected file.

#### Ancillary and Posttrial Care

At the conclusion of the trial, the active intervention will be made available to all participants in the control group.

## Results

Nationwide recruitment is currently underway with the aim of recruiting 600 people with type 2 diabetes. Recruitment will continue until October 2017.

## Discussion

### Principal Findings

Treatment of depressive symptoms in people with type 2 diabetes might help to improve short- and long-term social and vocational functioning. Internet-delivered psychotherapy is an effective treatment for depression in people with type 2 diabetes; however, few studies have focused on mild-to-moderately severe depressive symptoms where treatment need in diabetes patients is greatest. Rarely has social and vocational function been evaluated in studies of online depression treatments in type 2 diabetes; the effectiveness of a generic Internet-based program has not been studied in this patient group. This study will shed light on whether an Internet-delivered public health program has the potential to reduce unmet treatment need and lessen the personal and societal impact of mild-to-moderately severe depressive symptoms within the context of type 2 diabetes.

### Limitations

Trials of self-guided interventions frequently report high rates of attrition, including study dropout (ie, questionnaire noncompletion) and/or disengagement from the program [38]. Study attrition introduces selection bias and potential misrepresentation of treatment effects. To reduce the impact of actual participant dropout, we will recruit a substantially larger sample than is required on the basis of our sample size calculation. To minimize potential study dropout, we will utilize a combination of strategies that have been shown to positively impact retention in previous trials, including email and telephone prompts and reminders [39], recompense for questionnaire completion [40], and activities aimed at keeping the study “front of mind” and engaging for older participants [41,43]. In addition, so that questionnaire completion is not contingent upon program use, participants will access the study questionnaire outside the intervention (ie, via a link sent to a nominated email account). To maximize program use, automated program reminders will be sent by email to participants at biweekly intervals [29].

Volunteer bias is another possible weakness of this study, as our recruitment techniques may yield a sample that is healthier [44] and more highly motivated to learn new skills and engage with self-guided therapy. Problems occur, for example, if participation in our study is reflective of better health and a broader personal commitment to self-improvement of diabetes outcomes and daily functioning, as this is likely to result in within-group changes that are larger than for a less motivated and more representative sample of type 2 diabetes patients. However, since myCompass is broadly available for self-referral and use whenever and wherever people choose, the recruitment processes are consistent with both the self-help nature of the intervention and its eventual use in the type 2 diabetes population.

### Conclusions

The personal and economic costs of comorbid type 2 diabetes and depressive symptoms are substantial. However, the development of Internet-delivered interventions offers a potential solution to mitigate these impacts [13-15]. myCompass is a broadly available and efficacious public health intervention that can be delivered at minimal cost [45]. It therefore presents itself as a potentially effective and timely option for reducing unmet mental health need and ameliorating the personal and societal impact of co-occurring depression and type 2 diabetes.

## Appendix

Multimedia Appendix 1 CONSORT-EHEALTH checklist V1.6.1 [23].

## Abbreviations

CBT: cognitive behavioral therapy
CONSORT-EHEALTH: Consolidated Standards of Reporting Trials of Electronic and Mobile HEalth Applications and onLine TeleHealth
GET.ON MED: GesundheitsTraining.Online Mood Enhancer Diabetes
GP: general practitioner
HbA1c: glycosylated hemoglobin
HREC: Human Research Ethics Committee
MMRM: mixed-models repeated measures
PHQ-2: 2-item Patient Health Questionnaire
PHQ-9: 9-item Patient Health Questionnaire
RCT: randomized controlled trial
SMS: short message service
SPIRIT: Standard Protocol Items: Recommendations for Interventional Trials
UNSW: University of New South Wales
WSAS: Work and Social Adjustment Scale
